# Use of urinalysis during baseline diagnostics in dogs and cats: an open survey

**DOI:** 10.1111/jsap.13567

**Published:** 2022-11-06

**Authors:** N. H. Gibbs, J. C. Heseltine, M. Rishniw, M. B. Nabity

**Affiliations:** ^1^ Department of Small Animal Clinical Sciences Texas A&M University College of Veterinary Medicine & Biomedical Sciences College Station TX USA; ^2^ Department of Veterinary Clinical Sciences Purdue University College of Veterinary Medicine West Lafayette IN 47907 USA; ^3^ Veterinary Information Network Davis CA USA; ^4^ Department of Veterinary Pathobiology Texas A&M University College of Veterinary Medicine & Biomedical Sciences College Station TX USA

## Abstract

**Objectives:**

To describe how veterinarians utilise and perform urinalyses for dogs and cats.

**Materials and Methods:**

A survey, developed and distributed through the Veterinary Information Network, enlisted veterinarians who perform urinalyses for dogs and cats. Participants were directed to question banks based on whether urinalyses were performed in‐house, by an outside diagnostic laboratory, or using an in‐house automated instrument. Participants using multiple methods were directed to questions that related to the chosen methods.

**Results:**

A total of 1059 predominantly first‐opinion clinicians from the USA and Canada completed the survey. Participants performed urinalyses much less frequently than blood work during a routine examination. The most common factors preventing participants from performing a urinalysis with blood work included clients' financial constraints, difficulty obtaining urine and lack of perceived diagnostic need. The most common reasons for submission to a diagnostic laboratory included efficiency, more trusted results and convenience. Speed of obtaining results was the most common reason for performing urinalyses in‐house. Of the participants who performed in‐house urinalyses, fewer always performed a manual sediment examination (79%) as compared with urine‐specific gravity (99%) and manual dipstick (87%).

**Clinical Significance:**

This survey documents that urinalysis is often not used in senior patients as recommended by recent clinical guidelines for dogs and cats which can result in decreased diagnosis and impaired management of subclinical disease. There is significant variability in urinalysis methods despite veterinary guidelines promoting standardisation, and this could lead to inaccurate results.

## INTRODUCTION

Veterinarians commonly perform urinalyses during the evaluation of their patients. It constitutes the third arm of the minimum database, along with a complete blood count (CBC) and chemistry panel. Routine urinalysis allows veterinarians to gain information about renal glomerular and tubular function, lower urinary system health and other body systems (Fry 2011, Callens & Bartges 2015). Urine‐specific gravity (USG) and serum biomarkers of glomerular filtration rate help evaluate renal function (Barsanti 2012). Urinalyses and chemistry panels synergistically provide a better understanding of patients' health. Some of the most common diseases in dogs and cats require urinalysis for diagnosis. For example, Banfield's State of Pet Health report found that cystitis and chronic kidney disease were two of the most common diagnoses in cats (Banfield Pet Hospital 2016). Cystitis affected over 2% of their cat population ranging from young adult to senior, and chronic kidney disease affected over 10% of their geriatric cat population. These conditions require urinalyses for complete and correct diagnosis.

As perceptions of the roles of companion animals in society change, clients and veterinarians alike increasingly prioritise prevention and early diagnosis of diseases. Recently, canine life stages guidelines have been established by the American Animal Hospital Association (AAHA) for primary care diagnostics and treatments to help veterinarians optimise medical practices (Creevy *et al*. 2019). Five life stages are established: puppy, young adult, mature adult, senior and end‐of‐life. The guidelines for baseline blood work and urinalysis are determined by age group. In three of these life stages (young adults, mature adults and senior animals), urinalysis constitutes a component of the recommendations to allow veterinarians to determine a dog's baseline values and follow trends overtime. Following trends allows veterinarians to diagnose occult and early stages of specific diseases, such as chronic kidney disease, which often occur without clinical signs early in the disease course, but require an early diagnosis to implement measures that might slow disease progression (Jacob *et al*. 2002, Jacob *et al*. 2005, Grauer *et al*. 2008, Littman *et al*. 2013, Cortadellas *et al*. 2014). Similarly, AAHA and the American Association of Feline Practitioners (AAFP) have developed guidelines for evaluating laboratory work during different life stages. In cats 7 years of age and older, urinalyses are recommended as part of wellness examinations to allow for early detection of disease and evaluate for trends in laboratory parameters that may be concerning. In young adults (1 to 6 years), urinalyses should be considered based on the individual patient (Quimby *et al*. 2021). These guidelines highlight the need to have a better understanding of how, why and what factors determine when veterinarians perform urinalyses.

Urinalyses are composed of four parts – physical characteristics, USG, chemical analysis using multi‐test semiquantitative dipsticks and sediment examination (Duncan & Prasse 1976). Dipstick analyses identify urinary protein, glucose, ketones, pH, bilirubin and heme, whereas sediment exams identify cells (*i.e*. leukocytes, erythrocytes, epithelial cells), casts, crystals and infectious organisms. Recently, automated urine dipstick readers and urine sediment analysers have been developed to perform urinalyses in‐house more efficiently and consistently (Ferreira *et al*. 2018, Hernandez *et al*. 2019). These new analysers have expanded the methods used for performing urinalyses in‐house.

Although the utility of urinalyses is well‐established, the methodology and use of urinalyses in small animal veterinary practice have not been documented. The purpose of this study was to establish the factors that determine when, why and how veterinarians perform urinalyses.

## METHODS

We created a survey to explore factors that contribute to how, why and when veterinarians perform urinalyses for dogs and cats. An e‐mail with a link to the survey was sent to member veterinarians of the Veterinary Information Network (Appendix S1). No incentives were provided for veterinarians to complete the survey.

The survey had two sets of questions with different focuses. The first set of questions focused on demographics, including practice type, geographic area, species seen in the practice, practice size and graduation year from veterinary school. The second set of questions focused on general urinalysis practices, such as how urine is obtained for urinalysis, urinalysis methodology and how often urinalyses are performed compared to CBCs and chemistry panels. Four categories about how urinalyses are performed were available: in‐house manual examination (in‐house exam), sent to an outside diagnostic laboratory (outside lab), in‐house using an automated dipstick reader (dipstick reader), and/or in‐house using an automated sediment analyser (sediment analyser). Based on this question, the participants were given a select question bank that applied to each of the methodologies they selected. For example, if a participant only performed urinalyses using outside labs, then they were directed to an applicable question bank. However, if a participant used both outside labs and in‐house exams, then the participant answered two question banks that included questions about both.

Participants that performed in‐house exams were asked several questions regarding how urinalyses are performed, by whom, their reasons for performing urinalyses in‐house rather than sending samples to outside labs, and whether they have considered using an in‐house automated analyser. Questions for participants that sent urinalyses to outside labs included how they store the urine, factors that determine why they send urinalyses to laboratories rather than performing them in‐house, what component of the urinalysis is the main limiting factor for performing urinalyses in‐house, and if they would consider using an automated in‐house instrument, if available. Disease entities that may have driven the selection of measured urine components were not queried in the study. Veterinarians who reported using an in‐house dipstick reader and/or sediment analyser were asked which type of analyser they use along with questions regarding sample preparation and storage. Participants using sediment analysers were asked questions focused on if and when they send samples to outside labs or perform in‐house exams instead of, or in addition to, the sediment analyser. Additionally, participants in this group were asked how often they review images generated by the analyser and what prompts them to review images or send them to a clinical pathologist for review.

Descriptive statistics were performed in this study to evaluate the different facets of the data generated from the survey. The participants were divided into categories depending on the method of urinalyses performed. The data were reported as percentages and medians. Figures were generated using Microsoft Excel.

### Ethics statement

This study was performed as a collaboration between Texas A&M University and Veterinary Information Network. The study was deemed not to be human research and was therefore exempted from IRB approval.

## RESULTS

### Demographics

A total of 1182 participants filled out the online survey between September and October 2019. Thirty participants who did not complete the survey were excluded. Seven participants who completed the survey were excluded because they reported they do not see patients directly and/or do not perform urinalyses for dogs or cats. Eighty‐six participants (7.5%) were from countries other than the USA or Canada or did not state their country and were excluded. Of the remaining 1059 participants, 918 were from the USA (87%) and 141 were from Canada (13%) (Table 1). The median year that the participants graduated from veterinary school was 2000 (range: 1961 to 2019; 1059 responses). Most participants described their practice as first opinion with exclusive care for dogs and cats (Table 1).

**Table 1:** Demographics of participants from the USA and Canada who completed a survey regarding canine and feline urinalyses

	Number of respondents
Practice type	
First opinion	963
Emergency	60
Shelter	19
Referral	11
Academia	6
Total	1059
Practice locale	
Rural	173
Suburban	595
Urban	281
Other	7
No response	3
Total	1059
Species	
Dog and cat only	746
Mixed	87
Mixed with exotics	226
Total	1059
Number of full‐time veterinarians	
1	279
2 to 5	629
>5	146
No response	5
Total	1059

### Performing the minimum database

Two‐thirds of all participants (68%; 725 of 1059) reported that they do not perform a urinalysis with every/almost every CBC or chemistry profile, most commonly because of financial constraints (n=452), because they do not think the urinalysis is warranted (n=386), and/or difficulty obtaining urine (n=372). This contrasts to 89% of respondents (945 of 1059) who stated that they would perform a urinalysis when the patient has indications for urine testing. The most common reasons for not testing in this situation are difficulty obtaining urine (n=93) and financial constraints (n=60).

The number of participants that often or always perform urinalyses with CBC and chemistry panels varied widely based on the age of the patients (Table 2). For patients less than 7 years old, sevenfold more participants perform CBCs and chemistry panels compared to urinalyses. However, for patients over 7 years of age, only twice as many participants performed CBCs and chemistry panels compared to urinalyses.

**Table 2:** Relative frequency with which participants who completed a survey regarding canine and feline urinalyses perform a complete blood count (CBC), chemistry panel or urinalysis on patients as part of pre‐anaesthetic or annual testing

	Patients <7 years old, n (%)			Patients ≥7 years old, n (%)		
	CBC	Chemistry	Urinalysis	CBC	Chemistry	Urinalysis
Never	36 (3.4)	22 (2.1)	137 (12.9)	8 (0.8)	3 (0.3)	40 (3.8)
Rarely	142 (13.4)	80 (7.6)	463 (43.7)	32 (3.0)	13 (1.2)	167 (15.8)
Sometimes	278 (26.3)	265 (25.0)	371 (35.0)	108 (10.2)	75 (7.1)	354 (33.4)
Often	310 (29.3)	336 (31.7)	72 (6.8)	367 (34.7)	354 (33.4)	356 (33.6)
Always	293 (27.7)	356 (23.6)	16 (1.5)	544 (51.4)	614 (58.0)	142 (13.4)
Total	1059	1059	1059	1059	1059	1059

### Overview of methods used for urinalyses and urine collection

The majority of participants indicated that they perform urinalyses by multiple methods with most performing in‐house exams, followed by using outside labs (Table S1). The urinalysis method did not impact the median number of estimated urinalyses performed weekly (Table S1). For urine collection, most participants stated that they use cystocentesis (98.6%; 1044 of 1059) and free catch urine (owner‐obtained free catch: n=759, mid‐stream free catch: n=698, any free catch: n=609). The median percentage of time each method is used to collect urine was 50% for cystocentesis, 20% for mid‐stream free catch, 15% for any free catch, 10% for owner‐obtained free catch and 5% each for catheterization, owner‐obtained litter box, and “other.”

### In‐house manual examinations

Of the participants that use both in‐house exams and outside labs, the median percentage of time that they perform in‐house exams was 50% (range: 1 to 99%). For all participants that perform in‐house exams (n=728), common reasons for performing urinalyses in‐house (*versus* outside labs) were the ability to obtain rapid results (n=680), having the required equipment (n=305), availability of trained staff (n=323) and economics (n=263). Of 141 participants who chose “other” reasons, 75 explained that they believe the results are more accurate when done in‐house due to time delays causing artefacts with submission to outside labs, with crystal formation being a common concern.

**FIG 1:**
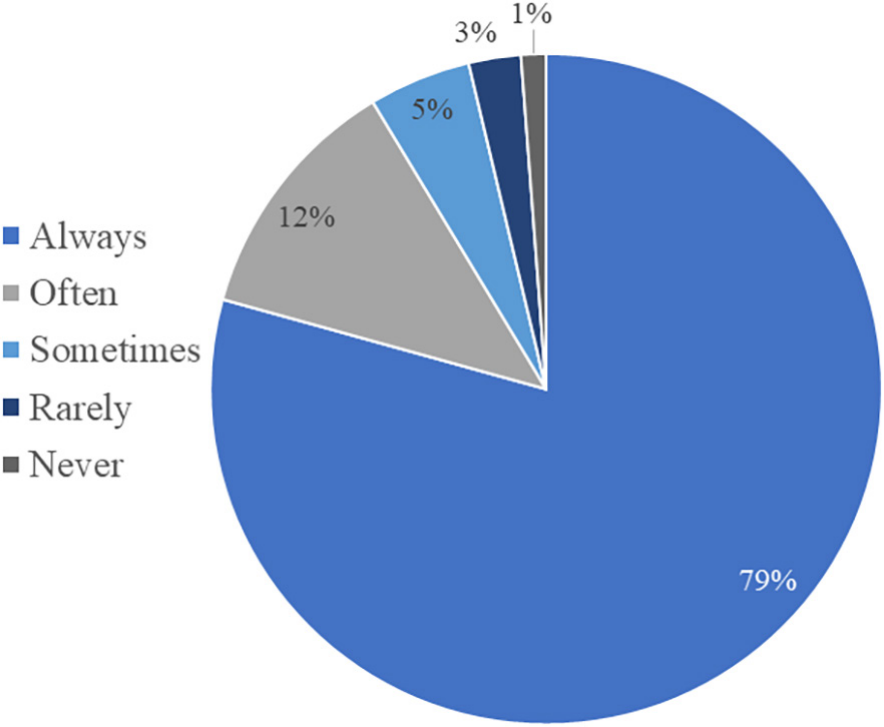
Relative frequency that participants without an automated sediment analyser perform urine sediment examinations as part of in‐house manual urinalyses in dogs and cats

Of participants that indicated they perform in‐house exams, 99% (720 of 728) always measure the USG when doing a urinalysis. Approximately 95% (694 of 728) of participants use a refractometer for USG measurement, whereas 0.1% (1 of 728) use urine dipstick alone and 4.4% (32 of 728) use both the urine dipstick and refractometer (Table S2), despite the inaccuracy of the urine dipstick for USG. In contrast, 87% of participants (495 of 570) who perform manual dipstick evaluation without dipstick readers stated they always perform a dipstick evaluation when doing in‐house exams, and 79% of participants that perform manual sediment examinations without sediment analysers always perform manual sediment examinations with their urinalyses (Fig. 1). Circumstances selected that would prompt a sediment examination for the participants who do not always perform these exams include lower urinary tract signs (80%; 94 of 117), abnormal urine appearance (71%; 83 of 117), white or red blood cells identified on the urine dipstick (45 and 42%; 53 and 49 of 117, respectively), and protein identified on the dipstick (44%; 51 of 117). Multiple participants commented that evaluation for crystals prompts sediment evaluation (n=10).

**FIG 2:**
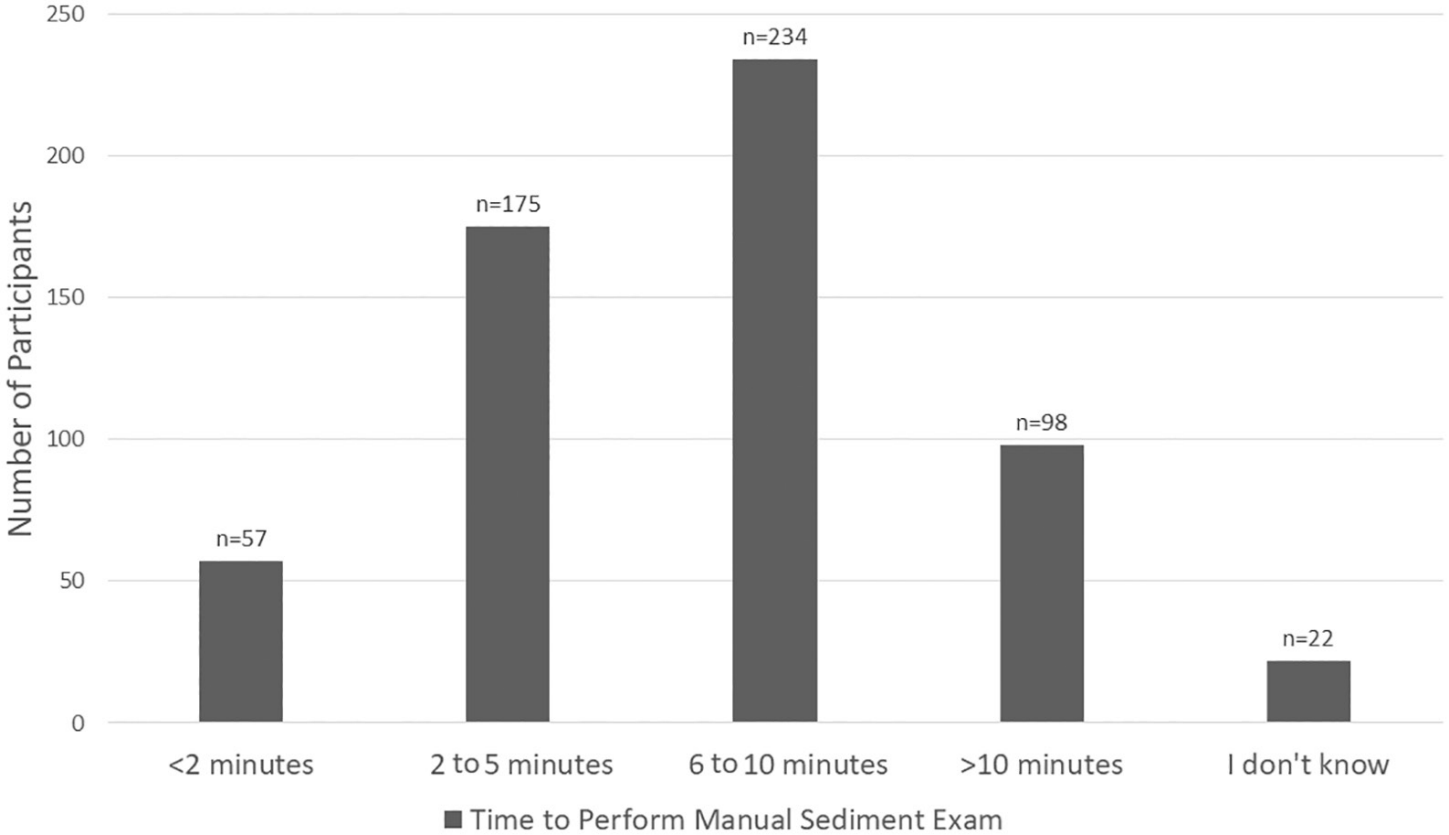
Time required to prepare and to perform manual urine sediment examinations in veterinary practices

Of the time categories provided, a plurality of participants indicated that manual sediment examination takes between 6 and 10 minutes to prepare and perform (Fig. 2). Practices most commonly use a combination of veterinarians and either licensed/certified veterinary technicians, or other trained staff, or both, to perform manual sediment examinations (43%; 256 of 595). Fewer use only veterinarians (30%; 178 of 595), only technicians (19%; 114 of 595) or only other trained staff (2%; 14 of 595), with the remainder using a combination of technicians and other trained staff. At the practices that use a combination of veterinarians and other employees (technicians and/or trained staff), veterinarians perform sediment examinations an average of 31 to 48% of the time.

Most participants that perform in‐house exams without sediment analysers complete both the dipstick and sediment evaluations within 30 minutes of collection (Fig S1). Few participants indicated that they refrigerate urine before in‐house analysis (n=51 for dipstick and sediment examination). Of those, 57% (29 of 51) and 37% (19 of 51) indicated that they perform the dipstick analysis or sediment examination, respectively, immediately or within 10 minutes of removal from the refrigerator. For manual dipstick analysis, 61% (346 of 570) of participants use unspun urine and 24% (139 of 570) sometimes use unspun urine and sometimes supernatant.

For participants that perform in‐house exams with or without sediment analysers, 84% of respondents centrifuge <3 mL or 3 to 6 mL of urine (345 and 267 of 728 responses, respectively). Additional information regarding how participants perform urinalyses are presented in Table S2.

**FIG 3:**
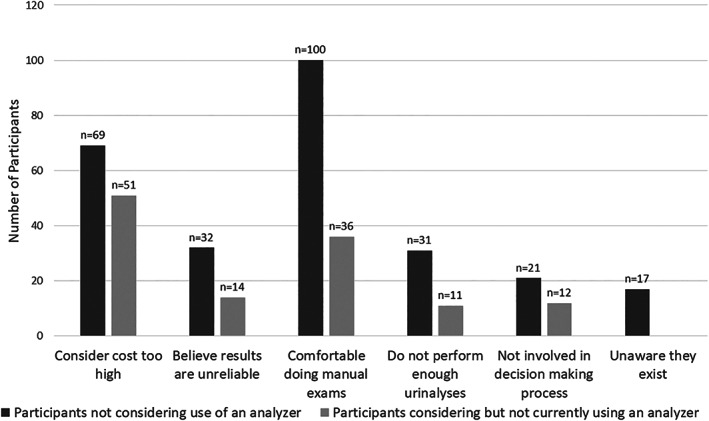
Number of participants who perform in‐house manual urine sediment examinations in dogs and cats grouped by reasons they do not use an in‐house automated urine sediment analyser

Of the participants who only perform in‐house exams, 35% (86 of 246) have considered obtaining a sediment analyser. Reasons for not obtaining a sediment analyser are described in Fig. 3.

### Outside diagnostic laboratories

Eighty‐nine percent (108 of 121) of participants that use outside labs store the urine in the refrigerator before collection by the courier. For these participants, the most common factors that contribute to sending urinalyses to outside labs are efficiency (105 of 121), trusted/reliable results (81 of 121), lack of expertise in urinalysis and/or sediment examination (79 of 121), and convenience (78 of 121). When asked which component of the urinalysis is the main limiting factor for performing urinalyses in‐house, 54 participants chose the sediment examination, two participants chose urine dipstick testing, and none chose USG. The time to analysis for outside diagnostic laboratories was not included in the survey.

Of participants who exclusively send urinalyses to outside labs, 28% (34 of 121) stated they would use a sediment analyser if one was available and 32% (39 of 121) stated they would not; 36% (44 of 121) of participants stated that they would consider using a sediment analyser. Participants indicated cost, accuracy and personnel time were the major deciding factors.

### In‐house automated instruments

Approximately one‐third of participants indicated that they use either dipstick readers, sediment analysers or both. However, only 7% of participants selected that they perform urinalyses exclusively using these methods (Table S1). Of the participants that reported using dipstick readers, 85% (275 of 325) use the IDEXX™ VetLab™ UA™ Analyzer and 13% (43 of 325) use the Abaxis™ VetScan™ Urinalysis Analyzer. One participant uses the Bayer™ Clinitek™. When asked the time between sample collection and performing the dipstick using a dipstick reader, 81% (264 of 325) of participants reported performing the analysis within 30 minutes.

**FIG 4:**
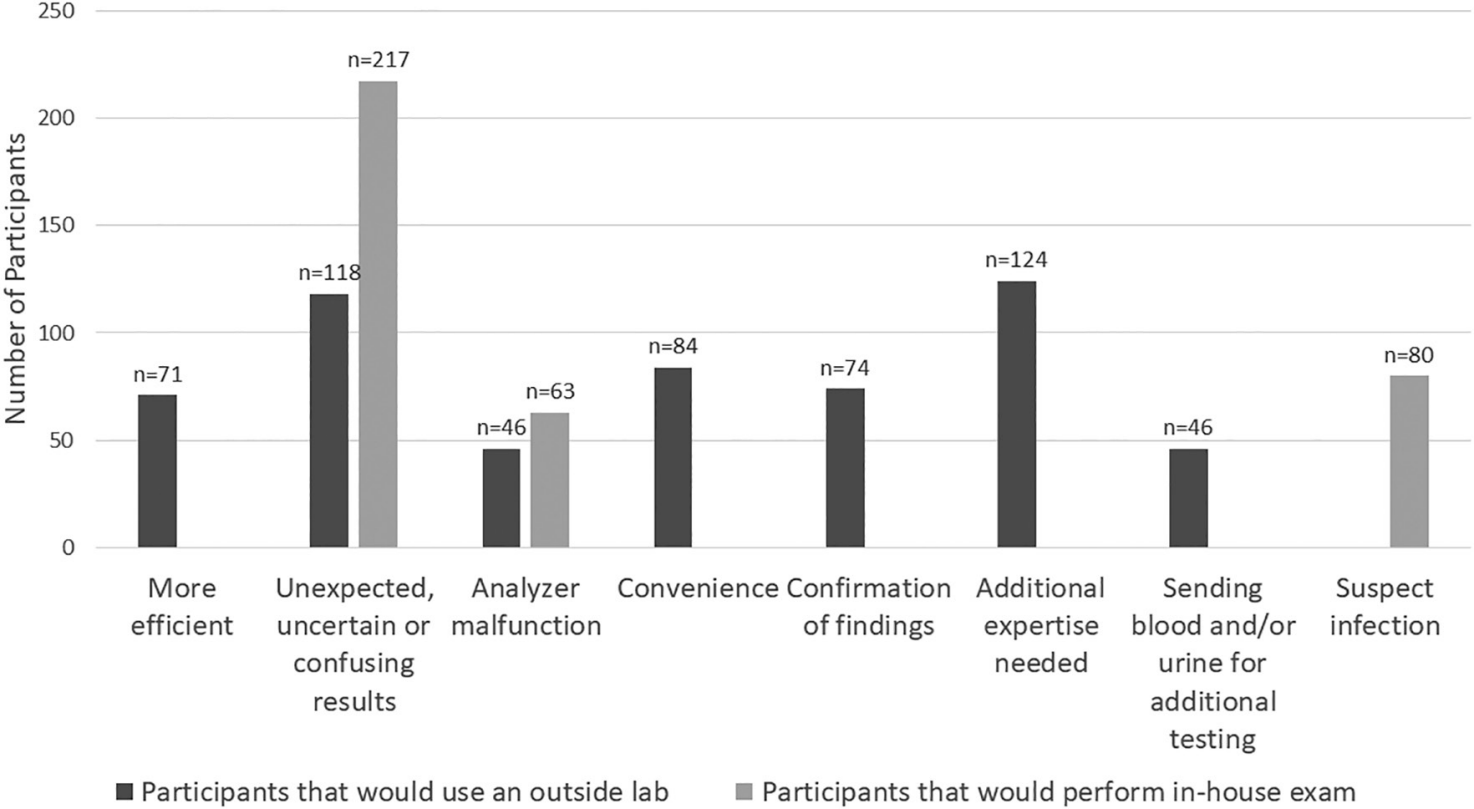
Number of participants with an automated urine sediment analyser grouped by reasons for performing a urine sediment exam manually in‐house (in‐house exam) or through an outside laboratory for dogs and cats

Of the participants who use sediment analysers, 89% (285 of 319) use the IDEXX™ SediVue™, whereas 8% (27 of 319) use the Abaxis™ VetScan™ Sediment Analyser, with the remaining participants being unaware of the brand they use. Of the participants who use sediment analysers, approximately two‐thirds also submit urine to outside labs. The most common factors leading to submission of urine to outside labs despite having a sediment analyser include the need for additional expertise for interpretation and unexpected/uncertain/confusing results from the automated analyser (Fig. 4). Another reason that the participants wrote‐in as an “other” response was that the urinalysis is part of package pricing with blood work or additional urine testing (*e.g*. culture or urine protein‐to‐creatinine ratio).

Eighty‐four percent (269 of 319) of participants who use sediment analysers indicated that they perform an automated sediment analysis on 90 to 100% of urine samples that undergo urine dipstick testing. Eighty percent (256 of 319) of participants stated that they perform the automated sediment evaluation within 30 minutes of urine collection. Twenty‐six participants indicated that they sometimes refrigerate samples before using the sediment analyser, and 18 (69%) run the urine sediment immediately or within 10 minutes of removal from the refrigerator. Of the participants who use sediment analysers, 79% (251 of 319) perform a manual sediment examination as a follow‐up or substitute for the sediment analysers, although 76% (191 of 251) of these respondents indicated that they rarely or sometimes do *versus* 24% (59 of 251) reporting that they often or always do this. The most common reasons for performing in‐house exams with a sediment analyser are described in Fig. 4. Additional reasons provided included exotic species patients, crowded samples or search for abnormal epithelial cells.

Of the participants who use sediment analysers, approximately 74% (236 of 319) reported often or always reviewing images selected by the analyser. The factors that prompt participants to review images included suspected bacteria (n=100); an unclassified crystal (n=68), cell (n=63) or cast (n=47); unknown material (n=58); confirmation of cells (n=52), casts (n=42) or the report in general (n=52); and/or the opportunity to learn (n=50). Most participants reported never (247 of 319) or rarely (n=62) sending images to a clinical pathologist for review.

## DISCUSSION

Our survey of first‐opinion small animal clinicians revealed that urinalyses are commonly excluded as part of the minimum database, even for older patients. Furthermore, participants who perform in‐house urinalyses omit examination of the urine sediment more often than other components of the urinalysis. Our survey suggests that clinicians in first‐opinion practice underutilize urinalyses in older patients. The results of our study also demonstrate the variability and lack of standardisation of urinalysis techniques in veterinary clinics despite published clinical guidelines (Gunn‐Christie *et al*. 2012, Arnold *et al*. 2019).

Two‐thirds of all participants reported that they do not perform a urinalysis with every or almost every CBC and/or chemistry profile. Even for patients 7 years or older, urinalyses are performed half as often as blood work. Despite AAHA canine life stages guidelines that recommend routine urinalyses for senior or geriatric dogs (Creevy *et al*. 2019), many participants omit urinalyses because they do not believe urinalyses are warranted. However, urinalyses are necessary to detect certain common diseases associated with ageing, such as glomerular disease (Macdougall *et al*. 1986). Studies have found that 13 to 19% of apparently healthy elderly dogs showed evidence of persistent proteinuria (Willems *et al*. 2016, Marynissen *et al*. 2017). Willems *et al*. (2016) reported that approximately 77% of dogs with proteinuria had evidence of hypertension with systolic blood pressure being 160 mmHg or above. Missing proteinuria and related hypertension can lead to progressive injury to multiple organ systems, including the kidneys and cardiovascular system, which are commonly associated with morbidity and mortality in older dogs and cats (Lees *et al*. 2005, O'Neill *et al*. 2013, Acierno *et al*. 2018). Therefore, clinicians who fail to routinely perform urinalyses likely fail to detect certain common diseases in dogs and cats at the frequency with which they occur.

The present study found that approximately 20% of participants who perform in‐house urinalyses do not always perform sediment examinations as part of the urinalysis. Of these participants, the most common factors contributing to performing a sediment examination were protein, white blood cells, or red blood cells identified on dipstick exam in addition to lower urinary tract signs. This is in line with the fact that an active urine sediment examination can negate the ability to interpret the protein present in the urine with regard to kidney disease (Bagley *et al*. 1991). Conversely, leukocyte identification by urine dipstick has little or no diagnostic value in dogs and cats (Callens & Bartges 2015). Therefore, limiting urine sediment examinations to only cases that yield a positive dipstick test result for white blood cells will likely fail to identify urogenital inflammatory conditions in some affected dogs or cats. In addition, without sediment examination, crystals and casts cannot be recognised, leading to missed indicators of disease.

Over 60% of veterinarians use multiple methods for performing urinalyses. Different strengths and weaknesses of each modality impacted veterinarians' decisions about which method to use. Strengths of in‐house manual examinations include faster and low‐cost results with fewer artefacts of time‐delayed samples, whereas weaknesses include the time it takes to perform this test and potential inaccuracies. In 30% of practices, veterinarians are the only personnel performing the urine sediment examinations. Even when other technical staff are utilised, veterinarians perform urine sediment examinations an average of one of three of the time. Given the average time of 6 to 10 minutes to prepare and perform urine sediment examinations, procedure time might limit veterinarians and technicians from performing urinalyses in‐house, or it might lead to incomplete analyses, with omission of the most time‐consuming component (*i.e*. the sediment examination). The strengths and weaknesses of sending urinalyses to outside labs are largely opposite of performing urinalyses in‐house. Some strengths of sending to outside labs include time efficiency and more trusted results due to more expertise with urine sediment examinations, whereas weaknesses of this method include increased costs and delayed analysis and results, which can affect clinical application.

Given that time constraint was one of the factors cited for why urinalyses are not done in‐house, increased efficiency afforded by using an automated urine sediment analyser could be helpful for increasing the consistency of performing a sediment examination with every urinalysis. For example, in our survey, 84% of the participants who use a sediment analyser perform sediment exams most of the time *versus* 79% of those who perform in‐house exams. Users of sediment analysers also reported performing the sediment evaluation within 30 minutes of urine collection more frequently (80%) than participants using in‐house exams (69%). The American Society of Veterinary Clinical Pathologists (ASVCP) quality assurance guidelines state that urinalyses should ideally be performed within 30 minutes of collection (Arnold *et al*. 2019); increasing the proportion of urinalyses within this window will help with consistency and accuracy of results. Short‐term refrigeration is acceptable; for example, sediment elements (*e.g*. leukocytes, erythrocytes) are stable for a maximum of 4 hours in the refrigerator (Arnold *et al*. 2019). However, many of those who refrigerate the sample perform the urinalysis within 10 minutes of removal from the refrigerator, in contrast to the recommendation to warm the sample to room temperature first.

Manual dipstick examinations were not considered the main limiting component for most participants. Moreover, the use of dipstick readers did not appear to increase the percentage of dipstick analyses performed within 30 minutes when compared to manual dipstick analysis (81 *versus* 79%, respectively). In addition, the present study found that 67% of participants that send urinalyses to outside labs did so because of the lack of expertise in performing urinalyses and/or sediment examination. Automated sediment analysers might allow participants to gain familiarity and experience with sediment examinations by reviewing the images provided, as supported by the participants who indicated that reviewing the images provides them with an opportunity to learn. Over time, this could allow veterinarians to feel more confident and educated about performing urine sediment examinations. However, there was some concern among participants about the accuracy of these analysers, and more studies are needed to determine the role of these new analysers in veterinary medicine.

Our survey highlights the variability in the protocol used for performing urinalyses and in particular, sediment examinations, in veterinary practices. In human medicine, there is less variability, largely due to more strict regulations regarding who can perform diagnostic testing. A urine sediment examination is considered a non‐waived test as part of the Clinical Laboratory Improvement Amendments (CLIA) (United States Food and Drug Administration 2020). The CLIA regulations are federal standards that are required to test human specimens for health assessment that must meet specific regulations. After these regulations were implemented, urinalyses were less commonly performed in doctor offices and more likely to be sent to outside diagnostic laboratories (Binns *et al*. 1998).

The ASVCP quality assurance guidelines for urinalyses provide an overview of preanalytical, analytical and postanalytical factors that are important in performing urinalyses (Gunn‐Christie *et al*. 2012, Arnold *et al*. 2019). These guidelines address many factors beyond time to analysis, including urine storage, handling and transport, sediment examination preparation, instrumentation and personnel knowledge. While our study examined several of these factors, others were out of the scope of the study (*e.g*. instrument quality control testing). Based on our survey, some of the more variable practices involved urine sediment preparation. Almost half of the participants use less than 3 mL of urine to prepare the urine sediment despite the recommendation that a standard volume of at least 3 to 5 mL be centrifuged (Barsanti 2012). Similarly, 63% of participants use less than the recommended 0.5 mL of supernatant to resuspend the sediment (Arnold *et al*. 2019). These reduced volumes can lead to errors in evaluating or interpreting the urine sediment as well as prevent comparisons from different samples. The ASCVP guidelines also recommend examination of a stained sediment smear or cytocentrifuged preparation for confirmation of bacteria as this results in higher specificity and sensitivity for detection of bacteriuria than wet‐mount alone (Arnold *et al*. 2019). However, only 19% of participants who perform in‐house exams state that they often or always use stain to confirm bacteria. Therefore, many participants could be misidentifying or missing bacteria as part of their sediment examinations.

We neither examine why deviations from recommended practice occur in veterinary clinics, nor why they occur so frequently; however, one possibility is a lack of awareness of such guidelines by most first‐opinion clinicians. The ASVCP guidelines were published in a specialty journal targeting mostly clinical pathologists (Gunn‐Christie *et al*. 2012, Arnold *et al*. 2019). Therefore, most first‐opinion clinicians would probably not encounter the guidelines in their reading. Further investigation is necessary to determine the reasons for the lack of standardisation and adherence to the guidelines in veterinary practices, since decreasing the variability in performing urinalyses may allow for increased accuracy and better interpretation of results.

The present study has some potential limitations, inherent with being a survey‐based study. Although a large number of participants were recruited for this study, the recruitment process involved veterinarians who are members of Veterinary Information Network, and this might introduce selection bias. Another potential limitation is that manual dipstick and sediment evaluations were included in the same category, which could have affected responses for manual sediment examinations, such as how often a sediment examination is performed. Other factors affecting the frequency of sediment examination could also have influenced participant responses if participants completed the survey considering patients in which a particular situation warranted a focused urine evaluation (*e.g*. urine glucose in a diabetic patient). Time to analysis after shipment to outside laboratories was also not queried but can significantly change the quality of the result. In addition, during data analysis and review of responses, some participants appeared to confuse automated urinalysis instruments and manual urinalysis examinations. When identified, these participants were excluded from analysis for the questions related to methodology. Because of the automated nature of the survey, computerised code was used to direct participants to specific question banks, and that is a potential source for error. There were eight participants who were funnelled to the wrong category as an error of the online survey protocol; these participants were excluded for the related questions. No other obvious discrepancies were identified on visual inspection of the data. Furthermore, given the large number of respondents, a minor number of participants being misdirected would likely have little or no impact on our findings.

In conclusion, the present study described the factors and methodologies of urinalyses in first‐opinion veterinary clinics. These results should be helpful for understanding the limitations for performing urinalyses, the variability that exists in practices, as well as opportunities for targeting methodologies to increase the efficiency and consistency of urinalyses.

## Author contributions

**Nicole Gibbs:** Conceptualization (supporting); data curation (equal); formal analysis (lead); methodology (equal); writing – original draft (lead); writing – review and editing (equal). **Johanna Heseltine:** Conceptualization (lead); data curation (supporting); formal analysis (equal); methodology (equal); project administration (equal); supervision (equal); writing – original draft (supporting); writing – review and editing (equal). **Mark Rishniw:** Data curation (lead); formal analysis (equal); methodology (lead); project administration (supporting); resources (lead); software (lead); writing – review and editing (equal). **Mary Nabity:** Conceptualization (equal); data curation (supporting); formal analysis (equal); methodology (equal); project administration (equal); supervision (equal); writing – original draft (supporting); writing – review and editing (equal).

## Supporting information


**Appendix S1.** Survey: urinalysis in veterinary practice.

**Fig S1.** Percentage of surveyed participants based on the amount of time urine is stored before examination when using manual dipstick, manual sediment, or automated sediment analyser for canine and feline urinalyses

**Table S1.** Number of surveyed participants and number of canine and feline urinalyses performed weekly by urinalysis methodology. The shaded areas indicate the methodologies used by that participant group

**Table S2.** In‐house urinalysis methodology for all participants that selected “in‐house manual exam” on a survey regarding canine and feline urinalyses
