# Supplementary material for: Use of urinalysis during baseline diagnostics in dogs and cats: an open survey
Source: J Small Anim Pract. 2022 Nov 6;64(2):88–95. doi: 10.1111/jsap.13567 (PMC10099574; doi:10.1111/jsap.13567)
Supplement: Supplementary file 1 — Appendix S1. Survey: urinalysis in veterinary practice. [file JSAP-64-88-s003.pdf]

Supporting Appendix 1. Survey: Urinalysis in veterinary practice

(untitled)

Page exit logic: Skip / Disqualify Logic
IF: #1 Question "Do you ever perform urinalyses on dogs and/or cats?" is one of the following answers ("No") **THEN:** Jump to page 20 - Thank You!

1. Do you ever perform urinalyses on dogs and/or cats? *

- Yes
- No

(untitled)

Page exit logic: Skip / Disqualify Logic
IF: #2 Question "What best describes your practice situation? If you work in a practice with more than one discipline, select the option that describes your personal employment status" is one of the following answers ("I do not see clients or patients directly") **THEN:** Jump to page 20 - Thank You!

2. What best describes your practice situation? If you work in a practice with more than one discipline, select the option that describes your personal employment status *

- First opinion practice - includes mobile practice
- Referral practice
- Emergency medicine
- Shelter medicine
- Academic practice
- I do not see clients or patients directly

(untitled)

Page exit logic: Skip / Disqualify Logic
IF: #3 Question "What best describes the types of animals you deal with in your practice? Select all that apply." is not one of the following answers ("Dogs","Cats") **THEN:** Jump to page 20 - Thank You!

3. What best describes the types of animals you deal with in your practice? Select all that apply. *

- ☐ Dogs
- ☐ Cats
- ☐ Horses
- ☐ Farm animals
- ☐ Exotics
- ☐ Birds
- ☐ Aquatic
- ☐ Other - Write In (Required)

*

(untitled)

4. What best describes your practice location?

- ○ Urban
- ○ Suburban
- ○ Rural
- ○ Other - Write In (Required)

5. How many full-time veterinarians (≥3 days/week) work in your practice?

- 1
- 2 to 5
- >5

6. How many part-time veterinarians (<3 days/week) work in your practice?

- 0
- 1 to 3
- >3

7. What year did you graduate from veterinary school?

2019
2018
2017
2016
2015
2014
2013
2012
2011
2010
2009
2008
2007
2006
2005
2004
2003
2002
2001
2000
1999
1998
1997

1996
1995
1994
1993
1992
1991
1990
1989
1988
1987
1986
1985
1984
1983
1982
1981
1980
1979
1978
1977
1976
1975
1974
1973
1972
1971
1970
1969
1968
1967
1966
1965
1964
1963
1962
1961
1960
Prior to 1960

General questions on performing UA

LOGIC Show/hide trigger exists.

8. Which of the following applies to your practice regarding urinalysis (i.e. urine dipstick and/or urine sediment exam)?
Select all that apply
*

- ☐ UA is performed by an outside laboratory
- ☐ UA is performed in-house by manual examination of urine dipsticks and sediment
- ☐ UA is performed in-house using an automated dipstick reader
- ☐ UA is performed in-house using an automated sediment analyzer

VALIDATION Min = 1 Max = 99 Must be percentage Whole numbers only Positive numbers only

LOGIC Hidden unless: (#8 Question "Which of the following applies to your practice regarding urinalysis (i.e. urine dipstick and/or urine sediment exam)?
Select all that apply
" is one of the following answers ("UA is performed by an outside laboratory") AND #8 Question "Which of the following applies to your practice regarding urinalysis (i.e. urine dipstick and/or urine sediment exam)?
Select all that apply
" is one of the following answers ("UA is performed in-house by manual examination of urine dipsticks and sediment","UA is performed in-house using an automated dipstick reader","UA is performed in-house using an automated sediment analyzer"))

9. What percentage of the time do you perform urinalyses (i.e. urine dipstick and/or sediment exam) in-house vs. an outside diagnostic lab? *

[ ] In-house

[ ] Send out to Lab

0 out of 100% Total

VALIDATION Must be numeric Whole numbers only

10. How many UA do you perform or send out in the average week?

11. Which urine collection method(s) do you use? Select all that apply. *

- [ ] Cystocentesis
- [ ] Catheterization
- [ ] Mid-stream free catch
- [ ] Any free catch
- [ ] Owner-obtained free catch
- [ ] Owner-obtained litter box
- [ ] Other - Write In (Required)

*

(untitled)

VALIDATION Min = 1 Max = 99 Must be percentage Whole numbers only Positive numbers only

LOGIC Hidden unless: ((((((#11 Question "Which urine collection method(s) do you use? Select all that apply." is not exactly equal to ("Cystocentesis") AND #11 Question "Which urine collection method(s) do you use? Select all that apply." is not exactly equal to ("Catheterization")) AND #11 Question "Which urine collection method(s) do you use? Select all that apply." is not exactly equal to ("Mid-stream free catch")) AND #11 Question "Which urine collection method(s) do you use? Select all that apply." is not exactly equal to ("Any free catch")) AND #11 Question "Which urine collection method(s) do you use? Select all that apply." is not exactly equal to ("Owner-obtained free catch")) AND #11 Question "Which urine collection method(s) do you use? Select all that apply." is not exactly equal to ("Owner-obtained litter box")) AND #11 Question "Which urine collection method(s) do you use? Select all that apply." is not exactly equal to ("Other - Write In (Required)"))

PIPING Piped Values From Question 11. (Which urine collection method(s) do you use? Select all that apply.)

12. What percentage of the time do you collect urine for UA by these methods?

0 out of 100% Total

Frequency of Urinalysis

LOGIC Show/hide trigger exists.

13. Do you perform a UA every time (or almost every time) that you perform a CBC/Chem profile? *

- Yes, every or almost every time
- No

LOGIC Hidden unless: #13 Question "Do you perform a UA every time (or almost every time) that you perform a CBC/Chem profile?" is one of the following answers ("No")

14. Why do you not perform a UA with every (or almost every) CBC/Chem? Select all that apply.

- ☐ Don't think it's usually warranted
- ☐ Increases the cost beyond the client's willingness to pay
- ☐ Difficulty obtaining urine
- ☐ UA results rarely change management decisions
- ☐ Other - Write In (Required) *

LOGIC Show/hide trigger exists.

15. Do you perform a UA when the patient has indications for UA testing? *

- ○ Yes, always
- ○ No, not always

LOGIC Hidden unless: #15 Question "Do you perform a UA when the patient has indications for UA testing?" is one of the following answers ("No, not always")

16. Why do you not perform a UA on all patients with indications warranting UA?
Select all that apply.

- ☐ Increases the cost beyond the client's willingness to pay
- ☐ Difficulty obtaining urine
- ☐ UA results rarely change management decisions
- ☐ Other - Write In (Required) *

(untitled)

Page exit logic: Skip / Disqualify Logic
IF: #8 Question "Which of the following applies to your practice regarding urinalysis (i.e. urine dipstick and/or urine sediment exam)?
Select all that apply
" is not exactly equal to ("UA is performed by an outside laboratory") **THEN:** Jump to page 13 - In-house testing

17. In a pre-anesthetic evaluation or annual exam of an apparently healthy patient **under 7 years** of age, how often do you perform the following? *

	Never	Rarely	Sometimes	Often	Always
CBC	○	○	○	○	○
Chemistry	○	○	○	○	○
Urinalysis	○	○	○	○	○

18. In a pre-anesthetic evaluation or annual exam of an apparently healthy patient **over 7 years** of age, how often do you perform the following? *

	Never	Rarely	Sometimes	Often	Always
CBC	○	○	○	○	○
Chemistry	○	○	○	○	○
Urinalysis	○	○	○	○	○

Outside Lab Testing

Page exit logic: Skip / Disqualify Logic
IF: #8 Question "Which of the following applies to your practice regarding urinalysis (i.e. urine dipstick and/or urine sediment exam)?
Select all that apply
" is not one of the following answers ("UA is performed in-house by manual examination of urine dipsticks and sediment","UA is performed in-house using an automated dipstick reader","UA is performed in-house using an automated sediment analyzer") **THEN:** Jump to page 20 - Thank You!

LOGIC Show/hide trigger exists.

19. Why do you choose to submit UA to a diagnostic laboratory, rather than performing it in-house?
Select all that apply

- ☐ More efficient use of my time
- ☐ Lack of expertise in performing UA
- ☐ Lack of expertise in performing sediment analysis
- ☐ Lack of appropriate equipment
- ☐ Convenience
- ☐ More trusted/reliable results
- ☐ Other - Write In (Required)

*

LOGIC Hidden unless: #19 Question "Why do you choose to submit UA to a diagnostic laboratory, rather than performing it in-house?
Select all that apply" is one of the following answers ("Lack of expertise in performing UA","Lack of expertise in performing sediment analysis")

20. Which component of the UA is the main limiting factor for not performing UA in-house?

- ○ Specific gravity measurement
- ○ Dipstick chemical analysis
- ○ Sediment examination

21. How do you normally store the urine prior to collection by the courier?

- ○ In a refrigerator
- ○ At room temperature
- ○ In a vehicle
- ○ Other - Write In (Required)

22. If a reliable and accurate urine sediment analyzer was available to you, would you perform urinalyses in-house?

- ○ Yes
- ○ No
- ○ Maybe - Please explain (Required)
- ○ I already use a urine sediment analyzer on occasion

In-house testing

Page exit logic: Skip / Disqualify Logic
IF: #27 Question "How often do you perform an in-house urinalysis using a **manual dipstick method** (**not** an automated urine dipstick analyzer)?" **THEN:** Jump to page 15 - Automated In-house Urinalysis

23. Why do you choose to perform some or all urinalyses in-house (as compared to sending the urine to a diagnostic laboratory)?
Select all that apply.

- ☐ More economical
- ☐ More rapid results
- ☐ Trained staff are available
- ☐ Have the equipment
- ☐ Other - Write In (Required)

*

LOGIC Show/hide trigger exists.

24. How do you **normally** store the urine prior to in-house urinalysis?

- ○ I usually run the UA immediately - no storage
- ○ Usually stored in a refrigerator
- ○ Usually stored at room temperature
- ○ Usually stored in a vehicle at vehicle temperature
- ○ Usually stored in a vehicle in a cooler
- ○ Other - Write In (Required)

LOGIC Show/hide trigger exists.

25. How often do you measure Urine Specific Gravity (USG) when performing in-house urinalysis? *

- Never
- Rarely
- Sometimes
- Often
- Always

LOGIC Hidden unless: #25 Question "How often do you measure Urine Specific Gravity (USG) when performing in-house urinalysis?" is one of the following answers ("Rarely","Sometimes","Often","Always")

26. How do you determine USG when performing urinalysis in-house? Select all that apply

- [ ] Urine Dipstick
- [ ] Refractometer

LOGIC Hidden unless: #8 Question "Which of the following applies to your practice regarding urinalysis (i.e. urine dipstick and/or urine sediment exam)?
Select all that apply
" is one of the following answers ("UA is performed in-house by manual examination of urine dipsticks and sediment")

27. How often do you perform an in-house urinalysis using a **manual dipstick method** (**not** an automated urine dipstick analyzer)? *

- Rarely
- Sometimes
- Often
- Always

Page exit logic: Skip / Disqualify Logic
IF: #8 Question "Which of the following applies to your practice regarding urinalysis (i.e. urine dipstick and/or urine sediment exam)?
Select all that apply
" is not one of the following answers ("UA is performed in-house using an automated dipstick reader") **THEN:** Jump to page 16 - (untitled)

28. How do you prepare the urine for **manual** urine dipstick testing?

- Always unspun urine (no preparation)
- Always on urine supernatant
- Sometimes unspun, sometimes supernatant
- I don't know
- Other - Write In (Required)

29. What is the average time between urine collection and UA when performing a **manual** urine dipstick analysis?

- <30 minutes (almost immediate)
- 30-60 minutes
- 1-3 hours
- 3-6 hours
- >6 hours
- I don't know

LOGIC Hidden unless: #24 Question "How do you **normally** store the urine prior to in-house urinalysis?" is one of the following answers ("Usually stored in a refrigerator")

30. How soon after removing urine samples from the refrigerator do you perform a **manual** urine dipstick analysis?

- Immediately
- <10 minutes
- <30 minutes
- <1 hour
- I don't know
- Other - Write In (Required)

LOGIC Show/hide trigger exists. Hidden unless: #8 Question "Which of the following applies to your practice regarding urinalysis (i.e. urine dipstick and/or urine sediment exam)?
Select all that apply
" is exactly equal to ("UA is performed in-house by manual examination of urine dipsticks and sediment")

31. Have you or your practice considered obtaining an automated urine analyzer and automated sediment analyzer? *

- Yes
- No

LOGIC Hidden unless: #31 Question "Have you or your practice considered obtaining an automated urine analyzer and automated sediment analyzer?" is one of the following answers ("No")

32. Why have you not considered obtaining an automated urinalysis system?
Select all that apply.

- [ ] Too expensive
- [ ] Don't believe the results are reliable
- [ ] Don't do enough UA to warrant it
- [ ] Comfortable performing manual exams
- [ ] Other - Write In (Required) *
- [ ] Didn't know they existed
- [ ] I am not involved in the decision process

LOGIC Hidden unless: #31 Question "Have you or your practice considered obtaining an automated urine analyzer and automated sediment analyzer?" is one of the following answers ("Yes")

33. Why have you not yet obtained an automated urinalysis system?
Select all that apply.

- [ ] Too expensive
- [ ] Don't believe the results are reliable
- [ ] Don't do enough UA to warrant it
- [ ] Comfortable performing manual exams
- [ ] Other - Write In (Required) *
- [ ] I am not involved in the decision process

34. What type of Automated Urine Dipstick Analyzer do you have?

- IDEXX VetLab Urine Analyzer
- Abaxis VetScan UA Analyzer
- Other - Write In (Required)

35. How do you prepare the urine for **automated** urine analysis?

- Always unspun urine (no preparation)
- Always on urine supernatant
- Sometimes unspun, sometimes supernatant
- I don't know
- Other - Write In (Required)

36. What is the average time between urine collection and UA when performing an **automated** urine analysis?

- <30 minutes (almost immediate)
- 30-60 minutes
- 1-3 hours
- 3-6 hours
- >6 hours
- I don't know

LOGIC Hidden unless: #24 Question "How do you **normally** store the urine prior to in-house urinalysis?" is one of the following answers ("Usually stored in a refrigerator")

37. How soon after removing urine samples from the refrigerator do you perform an **automated** urine analysis?

- Immediately
- <10 minutes
- <30 minutes
- <1 hour
- I don't know
- Other - Write In (Required)

(untitled)

LOGIC Show/hide trigger exists.

38. How often do you perform a **manual sediment examination** when performing in-house UA? *

- Never
- Rarely
- Sometimes
- Often
- Always
- I only perform an automated sediment examination

Manual Sediment Analysis

Page entry logic:
This page will show when: #38 Question "How often do you perform a **manual sediment examination** when performing in-house UA?" is one of the following answers ("Rarely","Sometimes","Often","Always")

Page exit logic: Skip / Disqualify Logic
IF: #38 Question "How often do you perform a **manual sediment examination** when performing in-house UA?" is one of the following answers ("Never","I only perform an automated sediment examination") **THEN:** Jump to page 19 - Automated Sediment Analyzers

LOGIC Hidden unless: #38 Question "How often do you perform a **manual sediment examination** when performing in-house UA?" is one of the following answers ("Rarely","Sometimes","Often")

39. Under what circumstances do you perform a manual sediment examination?
Select all that apply.

- ☐ Lower urinary tract signs
- ☐ WBC identified on dipstick or automated analyzer
- ☐ RBC identified on dipstick or automated analyzer
- ☐ Protein identified on dipstick or automated analyzer
- ☐ Urine has abnormal appearance (pigmented, cloudy)
- ☐ Other - Write In (Required)

__________ *

40. Who performs the manual sediment examination?
Select all that apply *

- ☐ Veterinarian
- ☐ LVT or CVT
- ☐ Other trained veterinary staff
- ☐ Other - Write In (Required)

*

Manual Sediment Examination

Page entry logic:
This page will show when: #38 Question "How often do you perform a **manual sediment examination** when performing in-house UA?" is one of the following answers ("Rarely","Sometimes","Often","Always")

Page exit logic: Skip / Disqualify Logic
IF: #8 Question "Which of the following applies to your practice regarding urinalysis (i.e. urine dipstick and/or urine sediment exam)?
Select all that apply
" is not one of the following answers ("UA is performed in-house using an automated sediment analyzer") **THEN:** Jump to page 20 - Thank You!

VALIDATION Min = 1 Max = 99 Must be percentage Whole numbers only Positive numbers only

LOGIC Hidden unless: (((#40 Question "Who performs the manual sediment examination? Select all that apply" is not exactly equal to ("Veterinarian") AND #40 Question "Who performs the manual sediment examination? Select all that apply" is not exactly equal to ("LVT or CVT")) AND #40 Question "Who performs the manual sediment examination? Select all that apply" is not exactly equal to ("Other trained veterinary staff")) AND #40 Question "Who performs the manual sediment examination? Select all that apply" is not exactly equal to ("Other - Write In (Required)"))

PIPING Piped Values From Question 40. (Who performs the manual sediment examination? Select all that apply)

41. What proportion of manual sediment examinations are performed by these individuals?

0 out of 100% Total

42. How soon after collection and chemical analyses do you **usually** perform **manual urine sediment** examinations?

- ○ <30 minutes
- ○ 30-60 minutes
- ○ 1-3 hours
- ○ 3-6 hours
- ○ >6 hours
- ○ Other - Write In (Required) ____________
- ○ I don't know

LOGIC Hidden unless: #24 Question "How do you **normally** store the urine prior to in-house urinalysis?" is one of the following answers ("Usually stored in a refrigerator")

43. How soon after removing urine samples from the refrigerator do you perform a manual urine sediment examination?

- Immediately
- <10 minutes
- <30 minutes
- <1 hour
- I don't know
- Other - Write In (Required)

44. How long, on average, does it take to prepare and perform a manual urine sediment examination?

- <2 minutes
- 2-5 minutes
- 6-10 minutes
- >10 minutes
- I don't know

45. How much urine do you usually centrifuge to obtain sediment for manual sediment examinations?

- <3 ml
- 3-6 ml
- 7-12 ml
- >12 ml
- We do not centrifuge prior to sediment exam
- I don't know
- Other - Write In (Required)

46. How much urine do you usually use to resuspend the sediment for manual sediment examinations?

- <0.5 ml
- 0.5-0.9 ml
- 1 ml
- >1 ml
- 20% of the centrifuge volume
- We do not resuspend prior to sediment exam
- I don't know
- Other - Write In (Required)

47. How do you usually mount the sediment for manual sediment examinations?

- Standard glass slide and cover slip
- Slide system specific for urine sediment analysis (e.g. UriSystem DeciSlide)
- I don't know
- Other - Write In (Required)

48. What sediment stain do you usually use for a wet-mount preparation when examining urine sediment?

- Sedi-Stain
- UriStain
- No stains are used
- I don't know
- Other - Write In (Required)

LOGIC Show/hide trigger exists.

49. If you observe bacteria-like elements on a manual sediment exam, do you use additional staining to confirm or characterize the suspect organisms?

- Never
- Rarely
- Sometimes
- Often
- Always
- I don't know

LOGIC Hidden unless: #49 Question "If you observe bacteria-like elements on a manual sediment exam, do you use additional staining to confirm or characterize the suspect organisms?" is one of the following answers ("Rarely","Sometimes","Often","Always")

50. What stain do you typically use to identify bacteria observed on the manual sediment exam?

- Diff-Quik
- Gram stain
- I don't know
- Other - Write In (Required)

Automated Sediment Analyzers

51. What automated urine sediment analyzer do you use?

- IDEXX Sedivue
- VetScan UA Sediment Analyzer
- I don't know the brand
- Other - Write In (Required)

VALIDATION Min = 1 Max = 100 Must be percentage Whole numbers only Positive numbers only

52. What proportion of in-house manual or automated biochemical urinalyses also undergo **automated sediment analysis**?

LOGIC Show/hide trigger exists.

53. Do you ever submit urine for sediment analysis to an outside laboratory, rather than performing an in-house automated analysis? *

- Yes
- No

LOGIC Hidden unless: #53 Question "Do you ever submit urine for sediment analysis to an outside laboratory, rather than performing an in-house automated analysis?" is one of the following answers ("Yes")

54. Why do you choose to send out urine for sediment analysis rather than performing an automated sediment analysis in-house?
Select all that apply

- ☐ More efficient to send out
- ☐ Analyzer malfunction
- ☐ Unexpected, uncertain, or confusing result from automated analyzer
- ☐ Convenience
- ☐ Confirmation of automated analysis
- ☐ Additional expertise needed for interpretation
- ☐ Other - Write In (Required) *
- ☐ All of the above

55. How soon after collection and chemical analyses do you perform **automated** urine sediment examinations?

- ○ <30 minutes
- ○ 30-60 minutes
- ○ 1-3 hours
- ○ 3-6 hours
- ○ >6 hours
- ○ Other - Write In (Required)
- ○ I don't know

LOGIC Hidden unless: #24 Question "How do you **normally** store the urine prior to in-house urinalysis?" is one of the following answers ("Usually stored in a refrigerator")

56. How soon after removing urine samples from the refrigerator do you perform an **automated** urine sediment examination?

- Immediately
- <10 minutes
- <30 minutes
- <1 hour
- I don't know
- Other - Write In (Required)

LOGIC Show/hide trigger exists.

57. Do you **ever** perform a manual sediment examination **after or instead of** using the automated sediment analyzer? *

- Yes
- No

LOGIC Show/hide trigger exists. Hidden unless: #57 Question "Do you **ever** perform a manual sediment examination **after or instead of** using the automated sediment analyzer?" is one of the following answers ("Yes")

58. How often do you follow up or substitute an automated urine sediment analysis with a **manual** urine sediment examination?

- Always
- Often
- Sometimes
- Rarely

LOGIC Hidden unless: #58 Question "How often do you follow up or substitute an automated urine sediment analysis with a **manual** urine sediment examination?" is one of the following answers ("Often","Sometimes","Rarely")

59. When and why do you do this?

- [ ] Unexpected, uncertain or confusing results on the automated sediment analyzer
- [ ] Analyzer malfunction
- [ ] When I suspect an infection
- [ ] Other - Write In (Required)

*

LOGIC Show/hide trigger exists.

60. How often do you review images selected by the automated urine sediment analyzer?

- ○ Never
- ○ Rarely
- ○ Sometimes
- ○ Often
- ○ Always

LOGIC Hidden unless: #60 Question "How often do you review images selected by the automated urine sediment analyzer?" is one of the following answers ("Rarely","Sometimes","Often")

61. What factors prompt you to review the digital images provided by the automated urine sediment analyzer?
Select all that apply.

- ☐ Unknown material
- ☐ Unclassified cell type
- ☐ Unclassified crystal
- ☐ Unclassified cast
- ☐ Suspected bacteria
- ☐ I do it as a learning opportunity even when the results do not require review
- ☐ Other - Please specify (Required) *
- ☐ Confirmation of any cell types, regardless of analyzer report
- ☐ Confirmation of any casts, regardless of analyzer report
- ☐ Confirmation of analyzer report, regardless of reported findings

LOGIC Show/hide trigger exists.

62. How often do you send images selected by the automated urine sediment analyzer to a clinical pathologist for review?

- ○ Never
- ○ Rarely
- ○ Sometimes
- ○ Often
- ○ Always

LOGIC Hidden unless: #62 Question "How often do you send images selected by the automated urine sediment analyzer to a clinical pathologist for review?" is one of the following answers ("Rarely","Sometimes","Often")

63. What factors prompt you to send digital images selected by the automatic sediment analyzer to a clinical pathologist for review?
Select all that apply.

- ☐ Unknown material in image
- ☐ Abnormal cell type in image
- ☐ Abnormal crystal in image
- ☐ Abnormal cast in image
- ☐ Suspected bacteria in image
- ☐ Other - Write In (Required)

__________ *
