# Supplementary figures and images for: Use of urinalysis during baseline diagnostics in dogs and cats: an open survey
Source: J Small Anim Pract. 2022 Nov 6;64(2):88–95. doi: 10.1111/jsap.13567 (PMC10099574; doi:10.1111/jsap.13567)

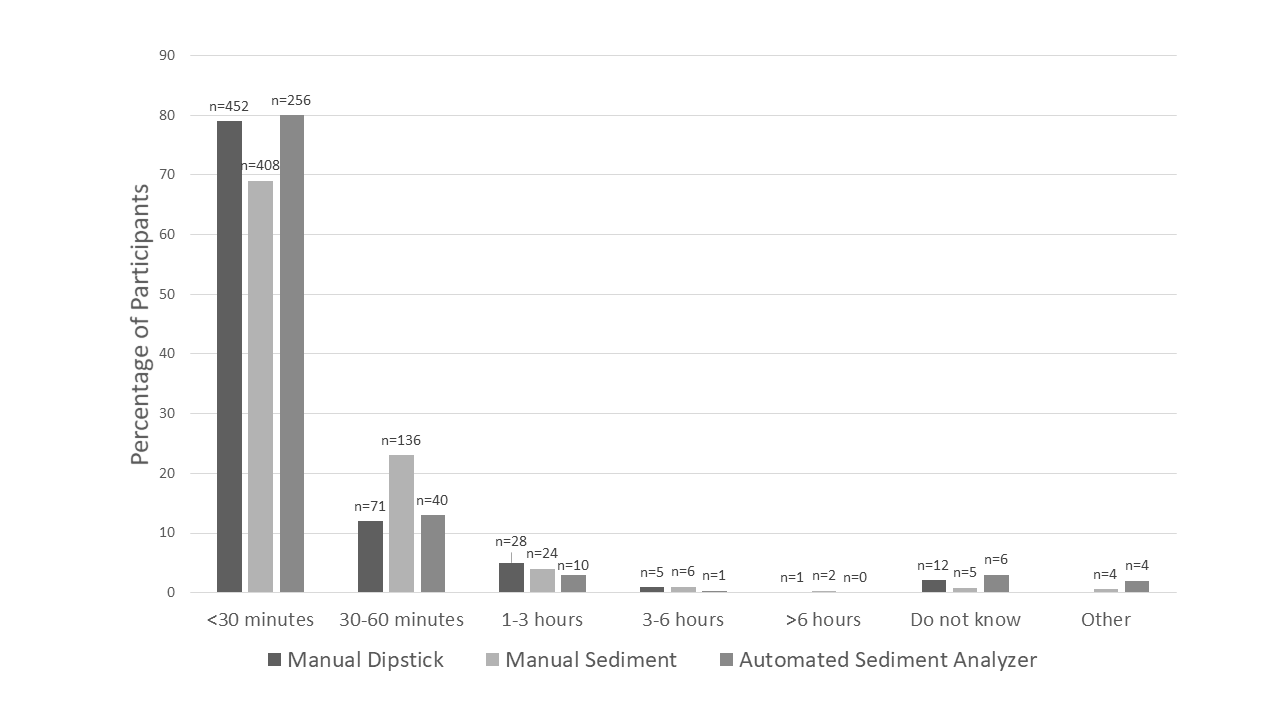

Supplement: Supplementary file 2 — Fig S1. Percentage of surveyed participants based on the amount of time urine is stored before examination when using manual dipstick, manual sediment, or automated sediment analyser for canine and feline urinalyses [file JSAP-64-88-s002.tif]
