# Supplementary material for: Use of urinalysis during baseline diagnostics in dogs and cats: an open survey
Source: J Small Anim Pract. 2022 Nov 6;64(2):88–95. doi: 10.1111/jsap.13567 (PMC10099574; doi:10.1111/jsap.13567)
Supplement: Supplementary file 3 — Table S1. Number of surveyed participants and number of canine and feline urinalyses performed weekly by urinalysis methodology. The shaded areas indicate the methodologies used by that participant group [file JSAP-64-88-s001.docx]

**Supporting Table 1:** Number of surveyed participants and number of canine and feline urinalyses performed weekly by urinalysis methodology. The shaded areas indicate the methodologies used by that participant group.

| **Combination category** | Combination of tests performed | | | | **Number of participants** | **Median Number of urinalyses performed weekly (range)** |
| --- | --- | --- | --- | --- | --- | --- |
|  | **In-house manual exam** | **Automated dipstick reader** | **Automated sediment analyzer** | **Outside diagnostic lab** |  |  |
| 1 | ✓ | ✓ | ✓ | ✓ | 73 | 10 (0-100) |
| 2 | ✓ | ✓ | ✓ |  | 39 | 7 (1-60) |
| 3 | ✓ | ✓ |  | ✓ | 28 | 9.5 (2-50) |
| 4 | ✓ |  | ✓ | ✓ | 13 | 10 (3-100) |
| 5 |  | ✓ | ✓ | ✓ | 67 | 10 (2-250) |
| 6 | ✓ | ✓ |  |  | 18 | 10 (2-50) |
| 7 | ✓ |  | ✓ |  | 8 | 5 (0-30) |
| 8 | ✓ |  |  | ✓ | 303 | 10 (1-100) |
| 9 |  | ✓ | ✓ |  | 76 | 9 (2-175) |
| 10 |  | ✓ |  | ✓ | 11 | 7 (2-25) |
| 11 |  |  | ✓ | ✓ | 13 | 10 (2-25) |
| 12 | ✓ |  |  |  | 246 | 6 (0-50) |
| 13 |  | ✓ |  |  | 13 | 5.5 (1-60) |
| 14 |  |  | ✓ |  | 30 | 10 (3-25) |
| 15 |  |  |  | ✓ | 121 | 7 (1-40) |
