# Supplementary material for: Use of urinalysis during baseline diagnostics in dogs and cats: an open survey
Source: J Small Anim Pract. 2022 Nov 6;64(2):88–95. doi: 10.1111/jsap.13567 (PMC10099574; doi:10.1111/jsap.13567)
Supplement: Supplementary file 4 — Table S2. In‐house urinalysis methodology for all participants that selected “in‐house manual exam” on a survey regarding canine and feline urinalyses [file JSAP-64-88-s004.docx]

**Supporting Table 2.** In-house urinalysis methodology for all participants that selected “in-house manual exam” on a survey regarding canine and feline urinalyses

| **Urine Storage Environment** | **Number of Respondents** |
| --- | --- |
| No storage/urinalysis performed immediately | 644 |
| Refrigerated | 66 |
| Room temperature | 10 |
| Other | 6 |
| Did not answer | 2 |
| Total: | **728** |
| **Time Urine Stored before Urine Dipstick Analysis** |  |
| <30 minutes | 574 |
| 30-60 minutes | 91 |
| 1-3 hours | 35 |
| 3-6 hours | 6 |
| >6 hours | 1 |
| Do not know | 20 |
| Did not answer | 1 |
| Total: | **728** |
| **Method of USG Measurement** |  |
| Refractometer | 694 |
| Urine dipstick | 1 |
| Both dipstick and refractometer | 32 |
| Did not answer | 1 |
| Total: | **728** |
| **Preparation of Urine for Manual Dipstick** |  |
| Always unspun urine | 447 |
| Sometimes unspun, sometimes supernatant | 173 |
| Always urine supernatant | 67 |
| Other | 10 |
| Do not know | 28 |
| Did not answer | 3 |
| Total: | **728** |
| **Urine Volume Centrifuged for Manual Sediment Exam** |  |
| <3 ml | 345 |
| 3-6 ml | 267 |
| 7-12 ml | 52 |
| >12 ml | 2 |
| Do not know | 39 |
| Other | 10 |
| Do not centrifuge | 3 |
| Did not answer | 10 |
| Total: | **728** |
| **Volume of Urine Resuspended for Manual Sediment Exam** |  |
| <0.5 ml | 459 |
| 0.5-0.9 ml | 50 |
| 1 ml | 20 |
| >1 ml | 9 |
| Do not resuspend | 77 |
| Do not know | 93 |
| Other | 9 |
| Did not answer | 11 |
| Total: | **728** |
| **Stain Used for Manual Sediment Examination** |  |
| No stains used | 210 |
| Sedi-Stain | 373 |
| UriStain | 22 |
| Other | 61 |
| Do not know | 53 |
| Did not answer | 9 |
| Total: | **728** |
| **Stain to Confirm Bacteria** |  |
| Always | 72 |
| Often | 66 |
| Sometimes | 124 |
| Rarely | 151 |
| Never | 290 |
| Do not know | 16 |
| Did not answer | 9 |
| Total | **728** |
| **Type of Stain to Confirm Bacteria** |  |
| Diff-Quik | 315 |
| Gram stain | 54 |
| Other | 23 |
| Do not know | 18 |
| Did not answer | 3 |
| Total: | **413** |
